# Biofeedback-Based Connected Mental Health Interventions for Anxiety: Systematic Literature Review

**DOI:** 10.2196/26038

**Published:** 2021-04-22

**Authors:** Mahra Alneyadi, Nidal Drissi, Mariam Almeqbaali, Sofia Ouhbi

**Affiliations:** 1 Department of Computer Science & Software Engineering College of Information Technology Al Ain, Abu Dhabi United Arab Emirates

**Keywords:** anxiety, biofeedback, systematic literature review, mental health, eHealth, mHealth, connected health, digital health

## Abstract

**Background:**

Connected mental health, which refers to the use of technology for mental health care and technology-based therapeutic solutions, has become an established field of research. Biofeedback is one of the approaches used in connected mental health solutions, which is mainly based on the analysis of physiological indicators for the assessment and management of the psychological state. Biofeedback is recommended by many therapists and has been used for conditions including depression, insomnia, and anxiety. Anxiety is associated with several physiological symptoms, including muscle tension and breathing issues, which makes the inclusion of biofeedback useful for anxiety detection and management.

**Objective:**

The aim of this study was to identify interventions using biofeedback as a part of their process for anxiety management and investigate their perceived effectiveness.

**Methods:**

A systematic literature review of publications presenting empirically evaluated biofeedback-based interventions for anxiety was conducted. The systematic literature review was based on publications retrieved from IEEE Digital Library, PubMed, ScienceDirect, and Scopus. A preliminary selection of papers was identified, examined, and filtered to include only relevant publications. Studies in the final selection were classified and analyzed to extract the modalities of use of biofeedback in the identified interventions, the types of physiological data that were collected and analyzed and the sensors used to collect them. Processes and outcomes of the empirical evaluations were also extracted.

**Results:**

After final selection, 13 publications presenting different interventions were investigated. The interventions addressed either primarily anxiety disorders or anxiety associated with health issues such as migraine, Parkinson disease, and rheumatology. Solutions combined biofeedback with other techniques including virtual reality, music therapy, games, and relaxation practices and used different sensors including cardiovascular belts, wrist sensors, or stretch sensors to collect physiological data such as heart rate, respiration indicators, and movement information. The interventions targeted different cohorts including children, students, and patients. Overall, outcomes from the empirical evaluations yielded positive results and emphasized the effectiveness of connected mental health solutions using biofeedback for anxiety; however, certain unfavorable outcomes, such as interventions not having an effect on anxiety and patients’ preferring traditional therapy, were reported in studies addressing patients with specific physical health issues.

**Conclusions:**

The use of biofeedback in connected mental health interventions for the treatment and management of anxiety allows better screening and understanding of both psychological and physiological patient information, as well as of the association between the two. The inclusion of biofeedback could improve the outcome of interventions and boost their effectiveness; however, when used with patients suffering from certain physical health issues, suitability investigations are needed.

## Introduction

### Background

Anxiety is the brain's way of reacting to stress and alerting to possible danger, which makes it an expected feeling in a person’s daily life, as it can be triggered by normal daily scenarios [1,2]. However, the persistence of this feeling might be an indicator that the person is suffering from an anxiety disorder. *Anxiety disorder* is an umbrella term that comprises various mental disorders characterized by excessive anxiety, tension, and fear that interfere with the person's daily life and interrupt the normal execution of daily tasks and activities [2]. There are 5 main types of anxiety disorders [3]: generalized anxiety disorder, obsessive compulsive disorder, panic disorder, posttraumatic stress disorder, and phobia. Most anxiety disorders affect women 2 times more than they affect men [3]. The causes of anxiety disorders are not clear and differ from one person to another based on many factors. Anxiety can be triggered by difficult life experiences, surrounding environment, and health behavior or can be caused by physical factors, such as overactive brain areas involved in emotions and behavior, genetics, and brain chemistry [4,5]. In addition to psychological symptoms, anxiety is also associated with many physiological symptoms including muscle tension, heartbeat issues, breathing issues, sweating, dry mouth, and headaches [1,3,5].

Connected mental health is the subfield of connected health that refers to the use of information and communication technologies for mental health care and includes all related areas such as mobile mental health, digital mental health, tele–mental health and e–mental health [6]. Connected mental health now plays a crucial role in the health care sector and contributes to improving the delivery of mental health care by providing novel, affordable, and easy-to-access solutions [6,7]. Technology has been included in mental health care in several forms including the exploitation of sensors for mental states’ detection and management [8], mobile apps for mental health care [9], and websites [10,11]. Use of technology has also facilitated access to many therapeutic solutions for anxiety disorders and mental health generally [7]. Examples include virtual reality (VR), computer- and internet-based cognitive behavioral therapy, and biofeedback [7]. VR is a 3D environment that can be either similar to or different from the real world. It allows the patients to interact with a specific environment based on the psychological issue and feared stimuli [12]. Computer-based cognitive behavioral therapy and self-rated mental health help evaluate the patient’s mental health via apps or websites by analyzing the user's answers to certain mental state assessment questions [13]. Patients can also use chatbots and online therapy services to communicate with mental health professionals and obtain a diagnosis [13,14]. Biofeedback-based mental health interventions aim to identify the patient’s mental state by monitoring body activities [15].

Biofeedback for mental health is based on measuring physiological changes associated with psychological states [16] to help monitor the body functions that are affected by the psychological reactions. Biofeedback-based interventions use and monitor different physiological factors, including heart rate, galvanic skin response (also known as electrodermal response), and respiration measurements. The main aim of biofeedback training is to provide patients with awareness and insight on their physiological changes, helping them better control those changes, and consequently, better control their mental state. Biofeedback has been shown to be one of the useful ways to help reduce the symptoms of anxiety disorders [17]. The physiological manifestations of anxiety make biofeedback useful in anxiety detection and treatment solutions.

Biofeedback could be useful for several mental health issues, such as stress, anxiety, hypertension, and depression [18]. Moreover, advances in technology have allowed biofeedback to become affordable, cost-effective, and easily used by practitioners as well as users [18]. The aim of this paper was to investigate the use of biofeedback in connected mental health solutions for anxiety disorders by conducting a systematic literature review. We investigated modalities of biofeedback use by identifying treatment approaches combined with it, types of sensors used in the interventions, and the physiological data collected and analyzed. In addition, we reviewed empirical evidence on the effectiveness of biofeedback-based interventions for anxiety from intervention outcomes.

### Related Work

Biofeedback is becoming one of the complementary and alternative medicine forms recommended by many doctors and therapists [16]. This section presents examples of literature addressing the adoption of biofeedback in treatment solutions for anxiety disorders and other mental issues.

Biofeedback in combination with psychotherapy was used for military medical providers suffering from anxiety, depression, and insomnia [19]. Psychotherapy helped reduce symptoms of anxiety and depression but could not improve insomnia issues. Yet when combined with biofeedback, the treatment was able to improve the sleep of the military medical providers [19].

A portable biofeedback device was integrated into clinical practice for patients with anxiety who were receiving cognitive behavioral therapy–based treatment [20]. Patients reported higher satisfaction with biofeedback-based treatment compared to that reported for other relaxation techniques such as meditation, yoga, and unassisted breathing. It was reported that biofeedback could be a promising treatment adjunct for disorders of autonomic arousal and could be easily integrated into treatment [20].

An exploratory review [21] investigated the efficacy of treating anxiety disorders in children and adolescents using biofeedback, cognitive behavioral therapy, and mindfulness combined with technological tools and programs, including serious games, web-based tools, apps, and internet-based tools. It reported that connected health interventions were found to be effective for anxiety management, as many studies reported they were as effective as traditional treatments.

Moreover, other studies [22-24] discussed the effectiveness of biofeedback in serious games for emotion regulation and mental health, generally. They reported that there was promising evidence for integrating biofeedback in serious games for managing anxiety [22-24]. Biofeedback has been reported as a successful approach to practice emotion regulation and was also found to improve performance on decision-making tasks in serious games [22]. Moreover, examples of existing biofeedback-based games have shown positive results for reducing depression and anxiety [24].

## Methods

### Overview

**Figure 1:**
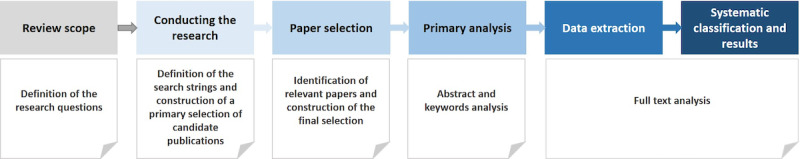
Review process.

We aimed to investigate the modalities of use of biofeedback in connected mental health solutions for anxiety disorders, as well as investigate the empirical evidence on such solutions. This study follows the quality reporting guidelines set out by PRISMA (Preferred Reporting Items for Systematic Reviews and Meta-analysis [25]). Figure 1 summarizes the review process.

### Research Questions

We attempted to answer the following 2 research questions: (1) What are the biofeedback-based connected mental health interventions for anxiety available in the literature? (2) How effective are biofeedback-based connected mental health solutions in treating anxiety? The first aims to identify the different treatment approaches that could be combined with biofeedback for the management of anxiety, as well as the different types of sensors and physiological information that could be used in biofeedback-based interventions. The second aims to identify patients’ interaction with the biofeedback treatments in connected mental health solutions and how beneficial it is to their case and level of anxiety.

### Research Method

The search for candidate papers was conducted in IEEE Digital Library, PubMed, ScienceDirect, and Scopus using the search strings presented in Table 1. The search strings included relevant terms and were formulated to identify a wide selection of candidate publications.

**Table 1:** Search strings.

ID	Search string
1	“biofeedback” AND “treatment” AND “anxiety” AND “disorder” AND (“e-health” OR “m-health” OR “digital health” OR “ehealth” OR “mhealth” OR “connected health” OR “technology”)
2	(“computer” OR “mobile”) AND “biofeedback” AND “anxiety” AND “disorder” AND “games”

### Paper Selection

**Figure 2:**
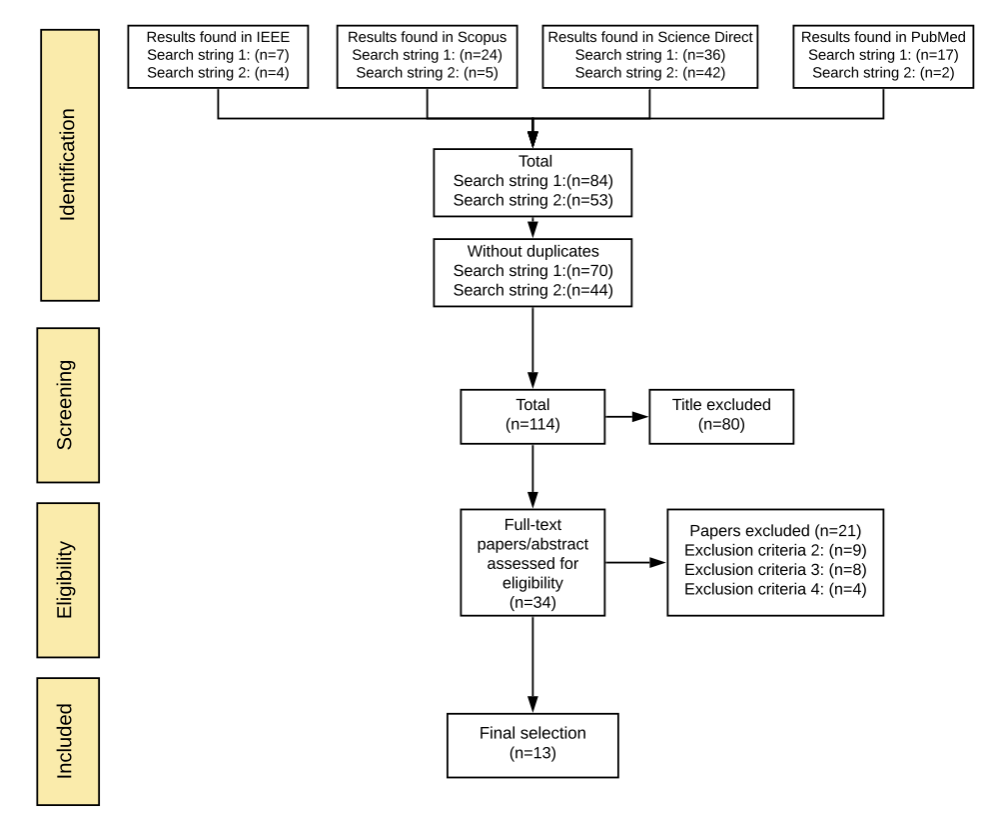
Selection process.

After the primary selection of candidate papers by applying the search strings to the digital libraries, duplicates were removed and the candidate publications were first analyzed by inspecting the titles then filtered based on a set of eligibility criteria to construct a final selection of relevant studies (Figure 2). Papers were selected based on the following inclusion criteria: studies that (1) addressed treating anxiety using biofeedback, and (2) combined information and communication technologies and biofeedback to treat anxiety. Exclusion criteria were any of the following: (1) publications that were not original research papers (eg, index or abstract); (2) studies that presented biofeedback as a treatment for general mental health or other issues, such as depression, but not anxiety; (3) studies that did not combine information and communication technologies and biofeedback to treat anxiety; or (4) studies that were not empirically evaluated (eg, reviews).

### Data Collection

Studies in the final selection were analyzed to extract relevant information. A data extraction form with predefined fields was developed in a spreadsheet (Microsoft Excel 2016). Data were extracted and categorized. While extracting data, we focused mainly on answering the research questions by identifying treatment approaches combined with biofeedback for anxiety management, the cohort groups addressed by the interventions, types of physiological data analyzed in the interventions, sensors used to collect the data, the processes of the empirical evaluations conducted on the interventions, and the outcome of empirical evaluations. Studies were then classified, mainly by grouping the interventions by cohort group and treatment approach.

### Synthesis

The synthesis method used in this study consisted of reading and analyzing selected studies, categorizing the data extracted from selected studies, and classifying the studies by enumerating the number of interventions for each data category. It should be noted that interventions including more than one treatment approach, more than one type of physiological data, or more than one type of sensor were counted in each category. The results are presented in figures and tables, because visualization of the results facilitated their analysis, and as a narrative summary describing the interventions and principal findings.

## Results

### Search Results

A total of 114 candidate publications were identified; however, only 13 studies met the eligibility criteria and were included in the final selection (Figure 2); 10 were published from 2011 to 2020, 2 were published in 2009, and 1 was published in 2002. The selected studies targeted people from different cohort groups: 8 of the interventions targeted mainly anxiety, while the rest addressed health issues associated with anxiety. In interventions that were identified, biofeedback was combined with different treatment techniques. Information on the publications, including targeted health issues and targeted cohort groups, are presented in Table 2, and information on processes and outcomes of the empirical evaluations are presented in Multimedia Appendix 1.

**Table 2:** Final selection.

Age category/reference		Year of publication	Cohort group		Condition	
**Adults**						
	[26]	2020	Patients with Parkinson disease		Anxiety	
	[27]	2019	Patients with rheumatic diseases		Pain and anxiety	
	[28]	2018	Patients with drug-resistant temporal lobe epilepsy seizures		Associated anxiety, stress, and depression	
	[29]	2014	Teachers and nurses		Psychological stress including anxiety	
	[30]	2009	Patients with generalized anxiety disorder		Anxiety	
**Young adults**						
	[31]	2018	Youth in residential care		Anxiety	
	[32]	2016	College students		Anxiety	
	[33]	2009	University students		Anxiety related to their studies	
**Children and adolescents**						
	[34]	2017	Children and adolescents with autism		Anxiety and performance	
	[35]	2016	Children		Risk of anxiety	
	[36]	2015	Pediatric patients		Pain and anxiety	
	[24]	2011	Children and adolescents		Anxiety	
	[37]	2002	Children		Migraine and anxiety	

**Figure 3:**
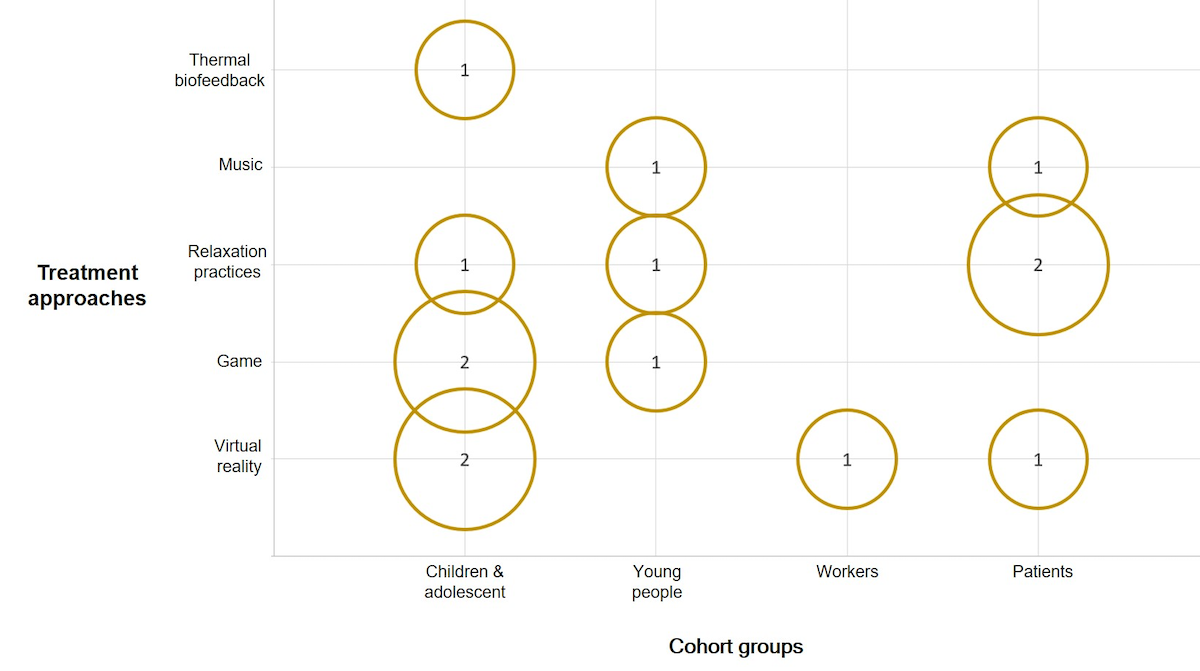
Bubble chart associating the treatment techniques with the target cohorts.

**Figure 4:**
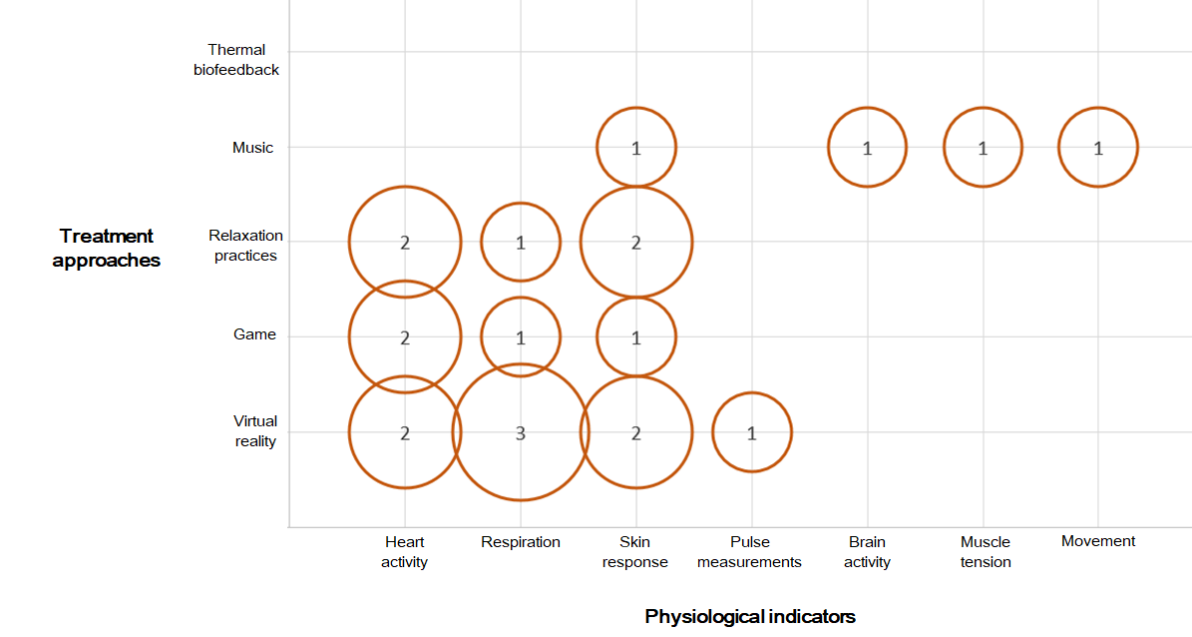
Bubble chart associating the treatment techniques with physiological indicators analyzed in the papers.

Identified treatment techniques included biofeedback with VR, games, and relaxation practices used with different cohort groups including children and adolescents, young adults, and patients (Figure 3). Children and adolescents were the age group most targeted by interventions. Figure 4 presents the association between treatment techniques and physiological data types. Data related to heart activity and skin response were used the most.

### Interventions for Adults

Of the 13 studies, 5 presented solutions for adults [26-30], 4 of which addressed patients [26-28,30]. Two of the solutions for patients were based on VR [27,30]. One was a VR-based biofeedback and guided meditation system for the management of pain and anxiety in patients with rheumatic disease [27] consisting of modules with and without respiratory biofeedback, monitored using a microphone. The user, when interacting with the VR environment, is instructed to breathe along to an oscillating pacer. Patients who used the intervention preferred the guided meditation module that did not include biofeedback over the module with biofeedback [27]. The other VR-based solution [30] was for the treatment of generalized anxiety disorder using VR and mobile phones, in which the user explored a virtual island by following a therapist-recorded narrative that included relaxation exercises. The user was also connected to biosensors that record physiological parameters including skin conductance, heart rate, and respiration. Some of the virtual experience elements were directly modified by the real-time heart rate variability of the user. Including biofeedback in the VR-based mobile intervention resulted in a higher decrease of anxiety than the VR-based mobile intervention without biofeedback [30].

Music therapy was also used with biofeedback; one intervention [26] consisted of an Ambulosono system for patients with Parkinson disease. The system is based on a method of musical gait training that rewards desirable gait behaviors with music play. The system comprised wireless headphones and a combination of a music player and a wearable sensor (iOS gyroscope and accelerometer) worn above the knee of the user to collect movement and step information. During gait biofeedback training, music is played when the user achieves a set stride length. Even though the data used in this intervention were not related to psychological status, the intervention had a secondary impact on patients’ levels of depression and anxiety [26].

A biofeedback system based on skin conductance used for the management of nonpharmacological epilepsy and associated anxiety, depression, and stress was proposed in [28]. The system presents the users with movies to trigger certain emotions that users were required to control [28]. While the users watched the films, skin conductance was measured and recorded using electrodes on the index and middle finger of the left hand of the participant. The study reported that skin conductance response was not related to changes in anxiety levels, which is contradictory to the findings of other studies in which skin conductance was used as an indicator of anxiety [38,39]. Yet this contradiction might be specific to the group targeted by the intervention. Even though the intervention reported a decrease in patients' anxiety and depression, it was suggested that this decrease reflects a nonspecific or a placebo effect of using the intervention [28].

For adult workers, particularly teachers and nurses [29], an intervention consisting of virtual scenarios with real-time monitoring was used for the management of the psychological state. The system put the user in virtual scenarios of stressful experiences and natural scenarios to learn certain relaxation techniques while the user was connected to cardiovascular belt and wrist biosensors to collect heart rate and heart rate variability. Data collected by the sensors were assessed by a decision support system that provided users with a real-time graphical representation of their current stress level. Some of the elements of the virtual experience were driven by the emotional status of the patient, measured by the biosensors. In this intervention, the combination of VR with biofeedback showed significantly better results than those of VR interventions without biofeedback or traditional treatments. The combination of VR and biofeedback showed better results in reducing anxiety when compared to traditional cognitive behavioral therapy treatment [29].

### Interventions for Young People

Of the 13 studies, 3 addressed mainly young people [31-33]. One of the interventions was a biofeedback video game called Dojo for anxiety and externalizing problems for young people in residential care [31]. The game promoted emotion regulation by providing tutorials on cognitive behavioral therapy–based relaxation techniques such as deep breathing, progressive muscle relaxation, positive thinking, and guided imagery. The game also included mini games that trigger different emotions such as fear, frustration, and anger and require the users to regulate their emotions using the techniques. User heart rate was monitored while playing the game through a biofeedback hardware and was displayed on the screen. Users were required to control their physiological reaction to succeed in the game, thus encouraging them to regulate their emotions [31].

Two interventions [32,33] targeted college students. One was based on music therapy and biofeedback treatment for anxiety [32]. The intervention provided the user with a relaxing music experience and judged the effect by measuring dynamic changes including skin resistance, as well as electroencephalography and electromyography indicators [32]. Another intervention provided biofeedback training to help university students overcome anxiety related to their studies [33]. The intervention is based on using a stress sweeper biofeedback device for the physiological measurements. Collected physiological indicators included heart rate and respiration. The biofeedback device helped guide participants while they practiced techniques to reduce anxiety related to their studies [33].

### Interventions for Children and Adolescents

Of the 13 studies, 5 presented solutions for children and adolescents [24,34-37]: 3 studies presented interventions based on games [24,35,36], including the programs *Freeze-Framer* and *Journey to the Wild Divine*, to reduce anxiety and depression. The programs were biofeedback-assisted relaxation training games that included cognitive behavioral therapy. The interventions used 2 to 3 electrodes to record momentary changes in heart rate variability and skin conductance levels. An example of a biofeedback relaxation activity offered by the programs is one in which users built a bridge—the user was required to relax to continue the construction of the bridge. As user breathing slows and tension decreases, the bridge is built; if the user experiences frustration or anxiety, the bridge disappears. After a continuous period of relaxation, the bridge is finished and the user can cross it [24]. Journey to the Wild Divine [36] collects heart rate variability and skin temperature of the users via sensors attached to the users' fingers. The program contains 15 levels, and completing one level requires meeting certain relaxation standards. Quick completion of a level is an indication of the ability of the patient to quickly regulate their physiological response and relax [36].

A VR biofeedback breathing game called DEEP [35] situates users in an underwater fantasy world, where they can move freely. The game is not based on levels or goals; rather, it provides personal breathing and meditation support by promoting diaphragmatic breathing through biofeedback. The game collects breathing data using a stretch sensor. A microcontroller interprets the sensor readings and sends data to the game, where it is used to change the user experience and manipulate the game elements. Slow and deep breathing allows players to move forward in the game, thus promoting diaphragmatic breathing. The intervention showed promising results; it was reported to decrease anxiety in a timely manner [35].

VR was also used in an anxiety-sensitive and performance-sensitive adaptive system for children with autism [34]. The system was composed of different modules including VR-based social communication, real-time physiological data acquisition, intelligent anxiety predictor, and strategy generator modules. The system captured photoplethysmography, electrodermal activity, and skin temperature signals to use as anxiety predictors. The system's purpose was to identify and quantify the user's anxiety level from real-time biomarkers, along with performance metrics during social communication simulations. The system adapts, progressing through different levels of tasks, based on the anxiety and performance metrics [34].

A system providing thermal biofeedback treatment to children suffering from migraine [37] included two types of thermal treatments, hand-warming biofeedback and hand-cooling biofeedback, to help the children deal with their migraine symptoms and anxiety. The treatments had different effects on migraines, yet did not affect anxiety levels [37].

### Empirical Evaluations

All 13 studies presented processes and outcomes of empirical evaluations. The empirical evaluations, in general, followed similar protocols: they included participants based on sets of eligibility criteria and divided participants into intervention and control groups who were wait-listed or received traditional treatment. Overall, the empirical evaluations yielded positive results. However, unfavorable outcomes were reported in some studies (Multimedia Appendix 1).

## Discussion

### Main Findings

**Figure 5:**
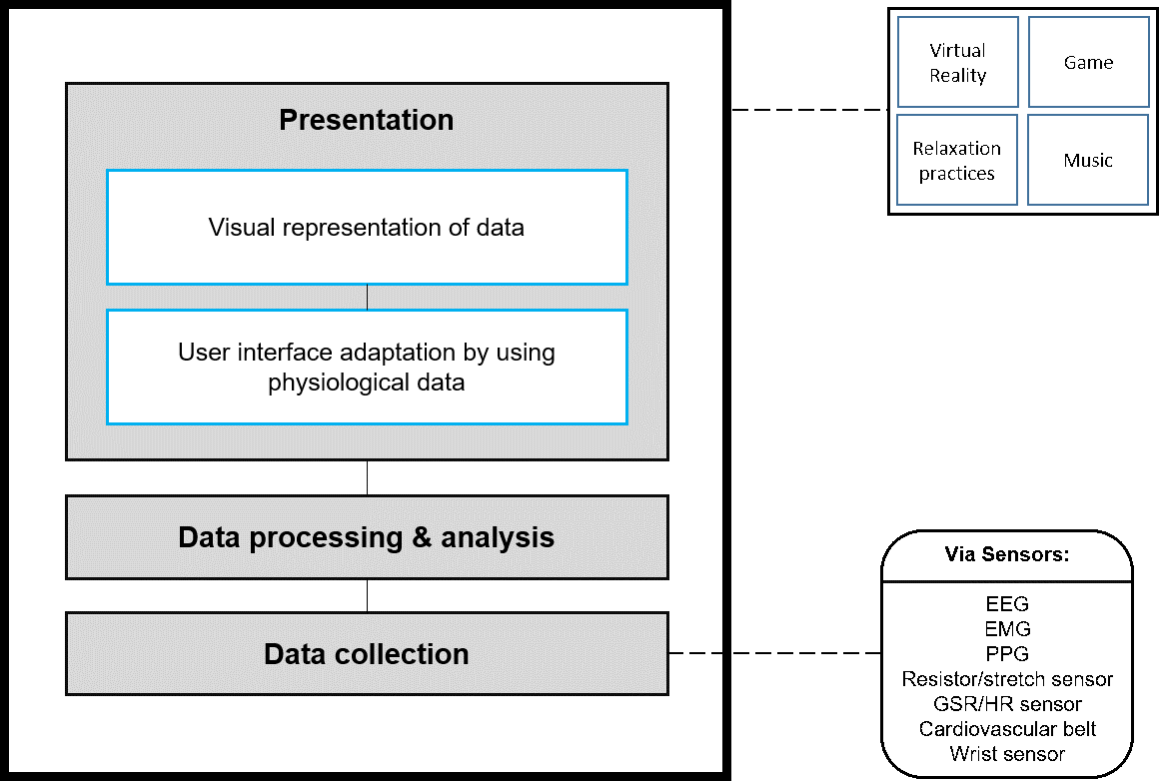
Layer diagram for a biofeedback-based system. EEG: electroencephalography; EMG: electromyography; GSR: galvanic skin response; HR: heart rate; PPG: photoplethysmography.

Interventions identified in this review used biofeedback with different treatment techniques, different sensors and physiological data, and for different purposes; however, all the interventions followed the same logic (Figure 5). The process begins with physiological data collection via sensors attached to the user. Physiological data are then processed and analyzed to generate a type of feedback from the system. In the interventions identified in this review, 2 main types of feedback were reported. Based on the readings of the physiological data the interventions either modified the user interface and experience or offered a visual presentation of the physiological changes.

### Biofeedback With VR and Games

Biofeedback was mostly found to be combined with VR and was found to increase the efficacy of such interventions [29,30]. The VR concept emerged in the 1980s and is based on transforming any real situation into a virtual experience. VR is based on the basic elements of generating images, presenting sensory information, and updating the displayed images based on the users’ position and orientation [40]. VR has the potential to create rich sensory experiences [41] and has been used for the management of different mental issues, such as the treatment of anxiety [42]. VR has been widely used for anxiety, by exposing patients to virtual situations triggering anxiety and teaching them how to deal with such situations. VR-based treatments have been used for different anxiety issues [43], including phobia management [44], mainly for the delivery of exposure therapy. VR facilitates exposure therapy exercises because they can be conducted in the therapist's office rather than in real-world phobic situations [44]. VR exposure has also been found to be effective for panic disorder [45] and in helping veterans with posttraumatic stress disorder [46].

Having issues interacting with the world is at the core of many psychological issues, including anxiety. VR could be a useful approach to face that issue, as it helps to simulate real-life experiences for the patients. Examples include helping manage phobia issues, which are characterized by intense fear and anxiety when interacting with certain elements, and posttraumatic stress disorder, in which patients have flashbacks of traumatizing experiences [42]. VR and biofeedback were also used with children [35], suggesting that it can be a children-friendly treatment. VR was used with children with multiple disabilities to familiarize them with the use of the wheelchair [47]. Moreover, VR has also been adopted into different types of games for purposes such as interactive entertainment, interactive training, and education [48]. The combination of VR with games has also been used in different medical settings as well, including medical training [49] and rehabilitation treatments [50].

Games have been coupled with biofeedback for mental health. Games for health can be classified as serious games, which mainly refers to games that do not focus on enjoyment, entertainment, and fun as their main purpose [51] but rather on elements such as education, training, and health improvement [52]. Quality games have been shown to influence behavior [53] and enhance concentration [54], as well as to facilitate learning and information retention [55,56]. Blending biofeedback with games has shown promising results. A computer biofeedback game based on animated gut imagery was used with people with irritable bowel syndrome and helped patients decrease their stress as well as mitigate symptoms related to their physical disorder [57]. Biofeedback-based games have also been used for physical issues management, for example, in balance training for people affected by chronic hemiplegic stroke, which showed positive results and was reported to be a feasible adjunct to conventional therapy [58].

Interventions including games mainly targeted the young generation [24,31] and have been shown to be effective. Young people are heavy users of technology, in general, and are the most familiar with gaming technologies, as millions of adolescents and young adults play video games and are the primary users of games [59,60]. Heavy use of technology and familiarity with games makes the young generation a suitable group for the use of game-based mental health care interventions. When introduced to VR interventions and games, biofeedback helped enhance the virtual and gaming experiences and encouraged user engagement, in addition to providing insight on user physiological and psychological states to help with the management of health issues. However, it must be noted that for such interventions to be helpful, user needs, experience, and preferences should be at the heart of their design [23,42].

### Biofeedback With Relaxation Practices and Music

Relaxation techniques and practices include somatic methods such as progressive relaxation, breathing, stretching, and physical exercises, as well as cognitive approaches including imagery, meditation, goal-directed visualization, and self-awareness [61]. These methods have been shown to be effective in mitigating anxiety and stress [61,62]. Biofeedback was coupled with such practices in some interventions [33,36] to inform users about the changes in their physiological measurements to help them better understand the association between their psychological and physiological indicators. Biofeedback inclusion was effective in the reduction of anxiety in these interventions [33,36].

Representing results with graphs, diagrams, animations, or other in order to deliver information for better understanding is used in education and is viewed as an effective teaching tool [63]. The same approach was used with patients to educate them about their health, which resulted in the patients being more satisfied, becoming more knowledgeable, and gaining understanding about their health issues [64]. In the case of anxiety, helping patients better understand and follow their case, the physiological changes associated with their mental issue, and treatment modalities can help them be more aware of the effect of anxiety on their bodies and help them be more accepting and trusting of the treatment applied, which might even influence the outcome of the treatment. Relaxation techniques were also used for pediatric pain management [33]. Relaxation techniques may be effective complementary therapies to pharmacologic techniques for pediatric pain, which can help reduce or even eliminate the amount of medication needed to treat the pain [65]. Relaxation approaches have also been used to manage other types of pain, including labor pain during childbirth [66], perioperative pain [67], and chronic pain [68].

Music with biofeedback was also among the relaxation techniques that showed positive results [32]. Music therapy has been shown to regulate both the physical and the mental health by affecting both the physiology and psychology of the person. Music can be combined with and included in different mental health management approaches and relaxation techniques [69] and can be used as a relaxation method on its own, as it was reported to be as effective as the progressive muscle relaxation method [70]. Because preferred or accepted types of music can be different in each community, when including music in psychological treatments, cultural consideration might be necessary [69].

Relaxation practices and music-based techniques have been widely adopted in technology-based interventions for anxiety, mainly in mobile apps [14]. Relaxation practices and music influence not only the psychology but also the physiology of our bodies. The inclusion of biofeedback could help improve mental care interventions based on those techniques. Biofeedback provides insight on physiological changes, which helps patients both assess and manage their psychological state.

### Biofeedback for Anxiety Associated With Other Health Issues

Anxiety was found to be common among patients of many physical disorders including patients with cancer [71] and people suffering from chronic obstructive pulmonary disease [72]. Anxiety is also common when undergoing medical procedures, such as magnetic resonance imaging [73]. Therefore, some interventions addressed symptoms of specific health issues and the anxiety associated with their prevalence [27,28,34]. Those interventions included solutions based on combining biofeedback with VR, which, in certain cases, showed unfavorable results [27]. This might be due to VR not being suitable for patients with health issues such as migraine, headache, seizure disorder, and vestibular abnormalities [43]. In addition, anxiety caused by the prevalence of specific health issues is generally impacted by the progress of the health issue itself, which might indicate that, in the interventions addressing other health issues, anxiety levels were impacted as a result of the change in the user’s health but not as a direct effect of the intervention on anxiety specifically [26,37].

### Implications

This review may be of interest to researchers, mental health interventions’ developers, and practitioners interested in the use of biofeedback in mental health management as it presents descriptions and analysis of 13 examples of biofeedback-based interventions for anxiety and different treatment approaches that could be combined with biofeedback, including VR, games, and music therapy. We also presented physiological indicators that could be exploited in biofeedback-based interventions and analyzed for anxiety management including heart rate measurements, respiration, and movement, which could be collected using different sensors including wrist sensors, cardiovascular belts, electroencephalography, and electromyography. The review showed different cohorts that could benefit from biofeedback-based interventions including children, patients, and workers. The interventions have generally yielded positive results in improving anxiety.

Use of biofeedback allowed better screening, understanding, and control of physiological factors during anxiety management interventions, improving outcomes and effectiveness; however, a need for additional investigation for certain health issues was highlighted.

It must be noted that this review might have some limitations: (1) the inclusion of other terms in the search string might result in additional publications and (2) searching Google Scholar might have resulted other relevant studies.

For future work, we intend to use the findings of this review to collaborate with mental health care professionals to create a biofeedback-based mobile app for the management of anxiety for young adults in United Arab Emirates.

## Appendix

Multimedia Appendix 1 Empirical evaluations.

## Abbreviations

PRISMA: Preferred Reporting Items for Systematic Reviews and Meta-analysis
VR: virtual reality
