# Supplementary material for: Biofeedback-Based Connected Mental Health Interventions for Anxiety: Systematic Literature Review
Source: JMIR Mhealth Uhealth. 2021 Apr 22;9(4):e26038. doi: 10.2196/26038 (PMC8103295; doi:10.2196/26038)
Supplement: Multimedia Appendix 1 [file mhealth_v9i4e26038_app1.docx]

## Multimedia Appendix 1

*Table 3 Empirical evaluations. Acronyms: Standard Deviation (SD), Parkinson Disease (PD), Virtual Reality (VR), Guided Meditation (GM), Respiratory Biofeedback (BFD), Autism Spectrum Disorder (ASD), Temporal Lobe Epilepsy (TLE), Skin Conductance Response (SCR), Diagnostic and Statistical Manual of Mental Disorders (DSM-IV), Visual Analog Scale (VAS), Social Responsiveness Scale (SRS), Social Communication Questionnaire (SCQ), Performance Sensitive (PS), Anxiety Sensitive (AS), Symptom CheckList-90 (SCL-90), Biofeedback-Assisted Relaxation Training (BART), Heart Rate Variability (HRV), Cognitive Behavioral Therapy (CBT), Generalized Anxiety Disorder (GAD), International Headache Society (IHS), VR and Mobile phone (VRM), VRM including Biofeedback (VRMB), Hand Warming Biofeedback (HWB), Hand Cooling Biofeedback (HCB).*

| **Ref** | **Participants** | **Age** | **Eligibility criteria** | **Procedure** | **Outcome** |
| --- | --- | --- | --- | --- | --- |
| [24] | 24 children and adolescent referred for treatment for anxiety | Between 9 and 17 years: Mean (12.88), SD (2.42) | Reporting symptoms of anxiety or having a diagnosis of an anxiety disorder | Participants were divided into 2 groups: an intervention group (n=12), and a waiting list as a comparison group (n=12). The intervention group received psycho-education and relaxation training, then started the game-based biofeedback sessions for a total of 8 sessions, 1 session per week | A decrease in anxiety and depression levels was identified in the post tests, as well as in comparison with the waiting list |
| [26] | 30 participants with mild to moderate PD | -Treatment group: Mean (67.5), SD (8.8)  -Control group: Mean (61.9), SD (8.0) | A clinical diagnosis of PD; the ability to walk for 15 minutes independently; being on stable medications; not suffering from active medical conditions that might interfere with safe participation; not suffering from severe cognitive impairment with functional consequences; not having spontaneous balance impairment or inability to walk | The experimental group received music contingent training for 12 weeks, where the music was adjusted online based on their performance. While, the control group received non-contingent music training for 6 weeks, and contingent training for the other 6 weeks | Both training groups showed improved mood and anxiety. However, no significant changes in cognitive measures |
| [27] | 20 patients from a rheumatology clinic | -BFD group: Mean (53.71), SD (15.94)  -GM group: Mean (51.9), SD (17.87) | Adult patients with chronic autoimmune disorders who were on a stable medication regimen; a score of at least 5 on the VAS for a minimum of 4 days within the prior 30 days | Participants were divided into 2 groups randomly. Group 1(n=10) received VR including GM, and group 2 (n=7) exposed to VR with BFD. Each treatment model lasted between 10 and 15 minutes | Significant decreases in pain and anxiety scales. However, the GM group recorded a noteworthy decrease in anxiety score while the VR using BFD group had no significant changes in anxiety scores |
| [28] | 30 drug-resistant TLE seizures patients | -Treatment group: Mean (35.27), SD (11.62)  -Control group: Mean (36.8), SD (9.814) | A diagnosis of drug-resistant TLE; age between 18 and 65 years; duration of epilepsy more than 3 years; seizure frequency at least 1 per month; stable medication one month before and during the study; and sufficient cognitive ability to keep a seizure diary | Participants were divided into 2 groups. The first group (n=15) received an SCR-based treatment in 12 sessions, during 24 months. The second group (n=15) was a waiting list | Significant improvements in seizure frequencies as well as depression and trait-anxiety were noticed in the treatment group, but not in the controlled group. A correlation was found between changes SCR and changes in seizures' frequencies but not between SCR changes and scores of psychiatric factors |
| [29] | 121 participants: teachers (n=61) and nurses (n=60) | -Experimental group: Mean (46.3), SD (7.7)  -Control group: Mean (42.9), SD (10.5)  -Waiting list: Mean (39.6), SD (9.7) | A high level of perceived stress; high level of relevance of stress for personal health; low level of self-efficacy related to stress management; no DSM-IV disorders; aged 25-60 years; no psychotherapy received for their psychological stress; no current psychiatric medications; no history of neurological diseases, psychosis, alcohol or drug dependence; no migraine, headache, or vestibular abnormalities | Participants were divided into 3 groups: an experimental group (n=40) receiving the VR and biofeedback treatment, a control group (n=42) receiving the traditional CBT treatment, and a waiting list group (n=39). The experiment lasted for 5 weeks with 2 treatment sessions per week | Both experimental group and control group exhibited a reduction in anxiety levels when compared to the waiting list. Yet the experimental group reported a significantly higher reduction of 12% when compared to the control group with a reduction of 5% |
| [30] | 12 patients with GAD | -VRMB group: Mean (41.25), SD (13.24)  -VRM group: Mean (48.5), SD (12.662)  -Waiting list: Mean (51.25), SD (9.845) | Diagnosis of GAD; age between 18 and 50; no psychotherapy treatment for GAD; type and amount of medication had to remain consistent during the experiment; no history of neurological diseases, mental retardation, psychosis, alcohol or drug dependence; no migraine, headache, or vestibular abnormalities; no pregnancy or breastfeeding | Participants were divided into 3 groups, including 4 participants each. Group 1 received the VRMB intervention, group 2 received VRM without biofeedback and group 3 a waiting list. Participants used the interventions for 8 sessions, bi-weekly | A higher decrease in anxiety after the VRMB treatment, compared to both VRM treatment without biofeedback, and the waiting list. |
| [31] | 37 youth in residential care | -Treatment group: Mean (13.67), SD (1.82)  -Control group: Mean (14.26), SD (1.94) | Elevated levels of both anxiety and externalizing problems; not being diagnosed with ASD or exhibiting psychotic symptoms | Participants were divided into 2 groups. Group 1 (n=20) received the Dojo game treatment in eight 30-min game-play sessions, and group 2 (n=17) received treatment as usual like CBT | Significant decrease in self-reported anxiety in the group using the Dojo game compared to the other group |
| [32] | 90 college students | _ | Anxiety factors more than three based on the SCL-90 | 3 groups were formed: a music therapy group (n=30), a music therapy combined with biofeedback group (n=30), and a control group (n=30). Treatments were delivered in a very calm environment with a soft lighting and comfortable seats to relax while turning on the music. Participants received the treatments for 10 sessions, 30 minutes each, once a week | Results showed improvements in both treatment groups. However, the biofeedback-based music therapy treatment had a significantly better impact on reducing anxiety. |
| [33] | 12 university students | _ | High anxiety levels and low academic performance | Participants were divided into 2 groups: an experimental group (n=6) receiving biofeedback training for 6 sessions and implementing the biofeedback-based treatments outside of the training sessions, and a control group (n=6) receiving no training | An increase in academic performance and a reduction in anxiety levels in the experimental group compared with the control group |
| [34] | 9 children and adolescents with ASD | Between 10 and 19 years | Attending a special needs school; DSM-IV diagnosis; cutoff T-score more than 60 in the SRS; and a cutoff score of 15 in the SCQ | Participants were exposed to a computer with VR-based tasks with biosensors placed on their thumb, middle, ring and index fingers. Participants were divided into 2 groups: group 1 received PS followed by AS tasks, and group 2 received AS followed by PS tasks | Most of the participants liked interacting with the system. Each level of the program had a different impact on the anxiety levels of the participants depending on the triggered emotions. When interacting with the AS tasks, a reduction in anxiety was noticed as the participants progressed in the levels |
| [35] | 86 children | Between 8 and 12 years: Mean (10.1), SD (1.4) | Attendees of the Cinekid Medialab multimedia exhibition for children | Participants played the game (DEEP) for 7 minutes in a comfortable environment | A significant decrease in self-reported state-anxiety was identified after playing the game for only seven minutes. |
| [36] | 66 pediatric patients | Between 8 and 25 years.  -Pain-only group: Mean (16.1), SD (4.2)  -Anxiety-only group: Mean (16.1), SD (5.7)  -Pain and anxiety group: Mean (16.2), SD (4.9) | Patients with medical conditions referred to the psychiatry consultation service for assistance with coping, who had comorbid psychiatric diagnoses | Participants were divided into 3 groups: pain only group, anxiety only group and pain and anxiety group. Over a duration of 18 months, each patient underwent at least 1 BART session of 45 minutes and had an HRV test | Improvements in pain and mood ratings. Patients with both pain and anxiety exhibited the greatest changes when compared to the other groups. |
| [37] | 36 children with migraine | Between 8 and 12 years | Between the ages of 7 and 17 years; qualified for an IHS diagnosis of migraine; no primary medical condition and a negative neurological examination; not taking daily preventative medication for headaches; and reporting an average of at least one migraine per week or 5 days per month with migraine | Participants were divided into 3 groups randomly: group 1 (n=13) received HWB treatment, group 2 (n=12) received HCB treatment, and group 3 (n=12) a control group. Participants received 4 sessions of treatment in a duration of 6 weeks | HWB group exhibited better clinical improvements in migraine compared to HCB groups. However, no significant psychological changes were identified |
